# Clinical Aspects of Bacterial Distribution and Antibiotic Resistance in the Reproductive System of Equids

**DOI:** 10.3390/antibiotics12040664

**Published:** 2023-03-28

**Authors:** Panagiota Tyrnenopoulou, George C. Fthenakis

**Affiliations:** Veterinary Faculty, University of Thessaly, 43100 Karditsa, Greece

**Keywords:** donkey, endometritis, horse, mare, mastitis, semen, stallion, *Streptococcus zoopidemicus*, therapeutics

## Abstract

Antibiotic administration is a standard therapeutic practice for the treatment of reproductive disorders of equids. This might lead to undesirable microbial imbalance and could favour the acquisition of antibiotic resistance. Therefore, it is imperative for clinicians to understand patterns of antibiotic resistance when considering and developing treatment regimes. Continued engagement of clinicians with novel alternative approaches to treat reproductive infections would be essential in order to address this rising threat within the One Health perspective. The objectives of the present review were to present the bacterial infections in the reproductive system of equids (horses, donkeys), to upraise the literature related to the issue of antibiotic resistance of bacteria causing these infections and to discuss the topic from a clinical perspective. Initially, the review summarised the various infections of the reproductive system of equids (genital system of females, genital system of males, mammary glands) and the causal bacteria, providing relevant information about horses and donkeys. Subsequently, the clinical therapeutics of these infections were presented, taking into account the significance of antibiotic resistance of bacteria as a limiting factor in treating the infections. Finally, approaches to circumvent antibiotic resistance in clinical settings were summarized. It was concluded that awareness regarding antibiotic resistance in equine reproductive medicine would increase, as we would recognise the multifaceted problem of resistance. Actions and initiatives within the One Health approach, minimizing the potential dissemination of resistant strains to humans and to the environment, with specific applications in medicine of equids should be appropriately instituted internationally.

## 1. Introduction

Antibiotic resistance poses a significant threat to global health and sustainability, and it is now regarded as a critical One Health issue. Antibiotic-resistant pathogens exist across the animal, human and environment niches [1]. In veterinary medicine, the increasing incidence of antibiotic resistance has been attributed to misuse of these agents and to reduced inflow of new antibiotics [2]. From the discovery of penicillin in 1928 to the ‘golden era of antibiotic discovery’ (in the 1950s to 1970s), antibiotics had been extensively used and have clearly contributed to increasing life expectancy. It is nevertheless noteworthy that in 1942, penicillin-resistant *Staphylococcus aureus* isolates were recovered, despite the short and limited use of the drug by that time [3]. Since then, various factors, among them, misuse and overuse of antibiotics in humans and animals (e.g., irrational prescription, self-medication, incorrect doses) have contributed to antimicrobial resistance becoming a significant threat in global health [4].

Antibiotic resistance of pathogens is a significant and increasing problem in veterinary medicine [5,6,7]. Comprehensive efforts to control antibiotic resistance of bacterial isolates from animals have been made, given the importance of the problem for animal health itself, as well as the animal–human interactions in a One Health approach [8].

With regard to equine medicine, a large amount of information on the importance of antibiotic-resistant pathogens in horses has been published [6,9,10,11,12,13]. These publications refer to research studies, whilst there is a lack of relevant reviews that would summarise and critically assess relevant studies from a clinician’s viewpoint [14,15,16]. It is also noteworthy that despite the use and importance of donkeys around the world, data regarding their reproductive disorders remain fragmentary [17] and, hence, these animal species have also been included in the present review, which thus refers to equids in a broader context.

The objectives of the present review were to present the bacterial infections in the reproductive system of equids (horses, donkeys), to upraise the literature related to the issue of antibiotic resistance of bacteria causing these infections and to discuss the topic from a clinical perspective.

## 2. Methodology

This narrative review includes primarily references published in journals cited in the Web of Science database (over 87% of all references cited); papers published in these journals have been refereed. Various search terms have been employed to identify relevant publications (e.g., ‘antibiotic resistance’, ‘antimicrobial resistance’, ‘horse*’, ‘donkey*’, ‘mule*’, ‘equid*’, ‘vaginitis’, ‘metritis’, ‘endometritis’, ‘orchitis’). Subsequently, the full papers have been retrieved through the websites of the respective journals. The papers were evaluated for suitability for citation and were included and cited if within the scope of the review. The remaining literature items refer to books and book chapters, which provide an overview of existing knowledge, theses, official documents of international organisations and information websites.

The median value of the year of publication of the references cited in the review is 2016, with the oldest reference cited date from January 1971 and the most recent one from March 2023, and an interquartile range for the year of publication of the references cited in the review of 12 years.

## 3. Bacteria in the Reproductive System of Equids

### 3.1. Bacteria in the Female Reproductive Tract

#### 3.1.1. Vagina

Knowledge of bacteria dominating vaginal microbiota is perennially important to comprehend their role in ascending equine reproductive diseases [18,19,20]. The vaginal vestibule is a major anatomical barrier that contributes to uterine defense against bacteria [21]. In the human reproductive medicine, there is evidence that the normal vaginal microbiome creates an acidic environment due to the dominance of *Lactobacillus* spp., which protects the reproductive tract from microbial ascension [22]. In contrast, *Lactobacillus* has been found to be less abundant in the vagina of mares, referring only to 0.18–0.37% of total bacterial numbers in that tissue [21,23]. In a study of healthy mares, in which the vaginal lactic acid bacteria were studied, *Lactobacillus equi*, *L. mucosae*, *L. pantheris*, *Enterococcus faecalis* and *E. faecium* predominated among these organisms [23]; the authors postulated that detection of these organisms suggested an intestine–vagina transference [23]. In a more recent study, the core vaginal microbiome of mares included Actinobacteria, Bacteroidetes, Firmicutes and Proteobacteria (at phylum level) and *Akkermansia* spp., *Arcanobacterium* spp., *Campylobacter* spp., *Corynebacterium* spp., *Fusobacterium* spp., *Kiritimatiaellae* spp., *Porphyromonas* spp. and *Streptococcus* spp. (at the genus level) [21]. No associations were found between the stage of the ovarian cycle and the composition of that microbiome [21]. Other researchers indicated that *Escherichia coli* predominated in the vagina, a finding that did not differ between mares in which insemination had been performed and unbred control animals [24].

The potential significance of *Leptospira* spp. should also be considered, as recent findings have indicated colonization of the vagina by *L. interrogans* serovars Australis and Bratislava of the organism (as shown by the detection of bacterial DNA in samples of vaginal fluid) [25,26]; the infection may lead in subclinical reduced-fertility of the affected mares [27], although results of relevant experimental studies have not provided conclusive evidence [28].

To note that variations in the vaginal microbiota may occur as the result of anatomical malformations (e.g., position and slope of external genitalia), which can lead to various disorders of the vaginal vestibule (e.g., pneumovagina, urovagina), use of irritant chemicals (e.g., antiseptics) during examination of the genital tract or artificial insemination, placement of intra-vaginal devices (for reproductive control), mating, vaginal injury or effects of antibiotics present in semen extenders. The above may lead to disturbance of the normal barrier and an imbalance of the normal vaginal microbiota [29,30].

It is noteworthy that, overall, the proportion of antibiotic-resistant bacteria recovered from the vagina of inseminated mares was higher than that among bacteria isolated from non-inseminated animals. Possibly, this might indicate a link with the exposure to antibiotics present in semen extenders [24].

With regard to female donkeys (jennies), previous studies have reported the recovery of various Gram-positive bacteria (*Bacillus* spp., *Corynebacterium* spp., *Lactobacillus* spp., *Staphylococcus* spp. and *Streptococcus* spp.) from vaginal samples collected from healthy animals. Among these, *Streptococcus equi* subsp. *zooepidemicus* isolates from individuals with reproductive disorders were found to carry a higher number of virulence factors genes than isolates from clinically healthy jennies [31,32,33]. Recent studies have revealed that the microbiome of the vagina of healthy jennies included mostly the phyla Proteobacteria (most abundant family: Moraxellaceae), Firmicutes (most abundant family: Lactobacillaceae), Bacteroidetes (most abundant family: Sphingobacteriaceae) and Actinobacteria (most abundant family: Corynebacteriaceae) [34].

#### 3.1.2. Uterus

Due to the proximity of the vagina to the uterus, vaginal microbiome studies could help elucidate the relationships between bacterial populations in these two organs. Initial studies on the bacteria present in the uterus of healthy mares included *E. coli*, *Klebsiella* spp., *Pseudomonas* spp., *S. equi* subsp. *zooepidemicus* and other *Streptococcus* spp. [18], organisms that have also been implicated as causal agents of abortions in mares and deaths of newborn foals [19]. More recently, the uterine microbiome of healthy mares has been investigated [35]; the researcher reported that, at phylum level, Bacteroidetes, Firmicutes, Verrucomicrobiota and Fusobacteriota, were more abundant, whilst at genus level, *Corynebacterium*, *Porphyromonas* and *Streptococcus* were more abundant. She also indicated that there were distinct differences to relevant findings in other animal species [35]. Various opportunistic microorganisms, e.g., Enterobacteriaceae, were also detected [35,36].

Endometritis is an important and common equine disorder, of great concern in stud medicine, as it is a primary cause of subfertility in mares [37] and has been diagnosed (in clinical or subclinical form) in 25% to 60% of mares with fertility problems [38,39,40]. It is the result of bacterial infection, precipitated by various factors, e.g., anatomical morphology of the genital tract and the perineal region, management variables and even animal breed [39]. The most commonly isolated bacteria from uterine swabs collected from mares with endometritis include *β*-haemolytic streptococci, *E. coli*, *Klebsiella* spp., *Pseudomonas* spp. and *Staphylococcus aureus* [20,41,42,43,44]. Other studies have implicated *Actinomyces* spp., *Bacillus* spp., *Corynebacterium* spp. and *Lactobacillus* spp. in the aetiology of the infection [38,40,45]. A more recent study highlighted also a potential role for *Chlamydia abortus* in endometritis [46]. Endometritis has been associated with recovery of bacteria, which are members of the normal microbiota of the uterus, e.g., *S. equi* subsp. *zooepidemicus*, from the deeper layers of the organ in long-standing cases of the infection [43,47,48].

Contagious equine metritis caused by the Gram-negative *Taylorella equigenitalis* is a notifiable venereal disease, associated with subfertility and spontaneous abortions in mares [49,50]. The infection is an officially listed disease by the World Organisation for Animal Health [51]. The infection was first diagnosed during an outbreak of acute metritis that occurred during the breeding season of mares in 1977 in Newmarket, United Kingdom [49] and, since then, it has been diagnosed in over 30 countries worldwide [52,53,54,55,56,57,58]. Since 2015, cases of contagious equine metritis have been confirmed in 17 countries worldwide (Belgium, Czech Republic, Denmark, Finland, France, Germany, Hungary, Poland, Slovenia, Republic of Korea, Republic of South Africa, Spain, Sweden, Switzerland, The Netherlands, United Kingdom and United States of America) [59]. The organism can be transmitted during natural mating or artificial insemination; it has an affinity for localization in the genital system of mares, usually in the clitoral fossa and clitoral sinus and occasionally in the endometrium, where it may also persist [60]. Such atypical infections by *T. equigenitalis* should be included in the differential diagnosis of equine subfertility, even in cases that the investigated mares had not been exposed to stallions or artificial insemination, of origin from countries where contagious equine metritis is prevalent [61].

During oestrus, when oestrogens dominate the endocrinological pattern, mares would clear bacterial contamination of the genital system efficiently. This is important, because, otherwise, the animals would be susceptible to endometritis. In this respect, many bacteria considered to be non-pathogenic might, nevertheless, cause genital infections. In contrast, mares with anatomical defects (e.g., poor conformation of the perineum, defects in the vestibulo–vaginal seal, cervical stenosis) or animals delaying uterine clearance, compromised immunity and/or exposed to venereal pathogens may fail to clear the bacterial load, as discussed above, which would potentially result in endometritis [30,62].

In sharp contrast to relevant results in mares, investigation of uterine infections of donkeys has been limited [33,34,63]. The uterine microbiome of healthy jennies was found to be composed mainly of the phyla Proteobacteria (most abundant family: Moraxellaceae), Firmicutes (most abundant family: Lactobacillaceae) and Actinobacteria (most abundant family: Corynebacteriaceae) [34]. However, in cases of endometritis, changes were evident in the frequency of abundance at family level, with Enterobacteriaceae, Lactobacillaceae and Sphingomonadaceae being the predominant families [34]. The abundance of the Sphingomonadaceae family has been postulated to be a consequence of using contaminated semen [34].

### 3.2. Bacteria in the Male Reproductive Tract

The semen of healthy stallions is considered to be bacteria-free, although it may become ‘contaminated’ during its passage through the genital tract at ejaculation [64]. Indeed, in those animals, the penis and the distal part of the urethra are frequently colonised by non-pathogenic bacteria [65,66]. Less frequently, pathogenic bacteria, e.g., *E. coli*, *Kl. pneumoniae*, *Ps*. *aeruginosa* and *S. equi* subsp. *zooepidemicus* may be recovered from those sites, often after mating [67,68]; Cerny et al. [67] indicated that a possibility of bacterial transfer to the stallion’s genital tract during mating could not be excluded.

Other pathogens responsible for infections in the genital tract of stallions include *Corynebacterium pseudotuberculosis* causing orchitis and epididymitis [69] and *Klebsiella pneumoniae* causing orchitis, epididymitis and seminal vesiculitis [70].

*Leptospira* sp. (*L. interrogans* serovars Bratislava and Copenhageni) was reported in the semen of stallions, which may play a role in sexual transmission of the pathogen [26,71]. The findings suggest that male equids should be confirmed to be *Leptospira*-free before the mating period, based on results of appropriate tests (e.g., findings of PCR testing in semen samples) [27].

There are limited data about the bacterial populations in male donkeys (jacks). Further to bacteria with confirmed pathogenicity for these animals (*S. equi* subsp. *zooepidemicus*, *Streptococcus equisimilis* and *S. aureus*), a variety of other organisms have been isolated from clinically healthy animals. These include Gram-positive (*Arcanobacterium* spp., *Bacillus* spp., *Corynebacterium* spp.) and Gram-negative bacteria (*Acinetobacter lwoffii*, *Oligella urethralis*, *Taylorella asinigenitalis*) [72].

*T. equigenitalis* is an obligate parasite, colonising the surface of the terminal urethra and its fossa in stallions [73], whence it may be transmitted to susceptible mares through direct contact of the semen [74]. It is noteworthy that infected stallions do not mount a humoral immune response and do not show clinical signs of fertility issues [73,75]. *T. asinigenitalis* is occasionally isolated from the genital tract of male stallions [76], as well as, notably, of jacks [77,78,79,80], and may create diagnostic problems due to identification confusion with *T. equigenitalis* [76,81,82]. The organism has been found to be pathogenic for mares, transmitted to them from jacks, whilst jennies have been recognised only as carriers [80].

### 3.3. Bacteria in the Mammary Glands

Bacteria were isolated from milk samples collected from over 40% of clinically healthy mares during the post-foaling period [83]. In general, mammary infections in female equids have been studied significantly less extensively than the respective infections in ruminants [84,85]. In cases of mastitis in mares, most of the bacteria isolated were identified as *Streptococcus* spp. [86,87], specifically *S. equi* subsp. *zooepidemicus* [83]. Other pathogens implicated in mammary infections are *Actinobacillus* spp., *Corynebacterium* spp., *E. coli*, *Klebsiella* spp., *Pseudomonas* spp., *Rhodococcus equi* and *Staphylococcus* spp. [83,85,88,89].

## 4. Therapeutics

### 4.1. General Considerations

In equids, antibiotic administration is important for the treatment of genital infections. Antibiotics are commonly used for the treatment of a variety of infections, including endometritis, metritis, mastitis in female and orchitis and epididymitis in male animals.

Selection of antibiotics must be based on rational principles and decision-making based on up-to-date scientific evidence after taking into consideration the drug, the bacteria involved and the animal. Equine clinicians have to consider a variety of issues in choosing the appropriate antibiotic regime. In most cases, at initiation of treatment, the pathogens involved (confirmed or presumed, as based on clinical findings and results of ancillary tests) in the infection must be taken into account. In a case of a seriously ill or immunocompromised equid patient, a patient with long-standing infection that has already received a broad range of antibiotics or a patient with a history of unsuccessful treatments, precise details are needed to facilitate selection of the optimal antibiotic. In such patients, an array of factors must be evaluated before prescription of the antibiotic.

Among these are results of bacteriological testing, which should include firm identification of causal agents and details of antibiotic susceptibility testing. A frequent reason for possible treatment failure is drug selection pressure, especially when these are ill-chosen and/or administered in suboptimal dose rates. This can lead, additionally to treatment failure, to survival of antibiotic-resistant bacterial populations and induction of antibiotic-resistance mechanisms to other isolates [90]. The recent findings of progressive increase in the MICs (minimum inhibitory concentration) of equine isolates of *S. aureus* underline the importance of this factor [91]. For *β*-lactam antibiotics, time necessary to reach concentrations over the MIC in the tissues of hosts is generally long [92]; then, their concentration must always exceed MIC throughout the treatment period for optimal bactericidal effect. In prescribing therapeutic protocols including *β*-lactam antibiotics, such pharmacodynamic relationships must be taken into account. Hence, for some infections, regimes including *β*-lactams may require the administration of these drugs up to four times daily [93].

Additionally, clinicians in choosing antibiotic for administration should take into account the One Health perspective and the World Health Organization classification of antibiotics into categories according to level of importance for treatment of human infections: critically important, highly important and important. In that case, possible alternatives should be preferred for administration.

In any case, potential adverse effects of the antibiotic considered for administration should also be taken into account [94]. These generally include patient- or dose-related adverse effects (e.g., nephrotoxicity caused by gentamicin or colitis induced by trimethoprim/sulphadiazine), as well as immunologic effects (e.g., hypersensitivity reactions or anaphylaxis, for example penicillin-induced reactions).

Examples of commonly applied antibiotic administration regimes against reproductive infections of equids are in Table 1 and Table 2.

### 4.2. Antibiotic Use in Female Genital Infections

Systemic administration of antibiotics, performed in combination with uterine lavage and administration of ecbolic agents, is a commonly accepted therapeutic protocol for endometritis, as it results in higher antibiotic concentrations in uterine fluid compared to intrauterine administration of the antibiotics [107,108]. In pregnant mares, antibiotic diffusion to the uterus is important for selecting the appropriate drug for administration in cases of placentitis [109,110]. Penicillin G and gentamicin [111] and enrofloxacin and trimethoprim–sulphonamides [110,112] have been found to achieve effective antibacterial concentrations in the uterus of mares. For the treatment of uterine infections, clinical studies have indicated that fourth generation cephalosporins, e.g., cefquinome, can have a broad spectrum of antibacterial activity against several uterine pathogens of mares, e.g., *E. coli*, *Pseudomonas* spp., *Staphylococcus* spp., *S. equi* [113,114,115]. *S. equi* genital infections do not respond well to trimethoprim–sulphonamide administration, even though the bacteria might have been found to be susceptible to these antimicrobials [116]. Additionally, in cases in which anaerobic bacteria have been isolated from uterine lavage samples, the administration of metronidazole can be considered [117,118].

Antibiotic administration is also recommended, along with non-steroidal anti-inflammatory drugs, in cases of retention of foetal membranes, which is deemed to be an emergency in equine clinical practice [119]. Non-steroidal anti-inflammatory drugs are beneficial as part of supportive treatment of mares with retention of foetal membranes because of their anti-inflammatory, analgesic and antiendotoxic effects. Flunixin meglumine is usually administered to mares post-partum (e.g., in cases of retention of foetal membranes, at a dose rate of 0.25 mg kg^−1^ thrice daily, intravenously), as it can minimise the adverse haemodynamic effects of endotoxaemia [120].

If intrauterine administration of antibiotics is to be selected, only non-irritating water-soluble antibiotics (e.g., amikacin sulphate, ceftiofur sodium, gentamicin sulphate, penicillin sodium, trimethoprim/sulfamethoxazole, with variations in the available commercial products between countries as per local licencing situation) should be used for successful outcome [38]. Relevant therapeutic protocols depend on the chronicity and severity of the infection and the bacteria involved. It is noted that this route of antibiotic administration can be used only post-partum or during oestrus, i.e., when the cervix is open. In any case, uterine lavage must be performed before infusion of antibiotics.

Uterine lavage by using normal saline after mating is often employed in stud farms because of its ability to increase the tone of uterine muscular layer and its effect in clearing microorganisms, spermatozoa, inflammatory cells and debris from the uterine lumen, as these have been found to contribute to early embryonic death in mares [121]. Use of lactated Ringer’s solution has also been advocated [122], whilst the use of iodine povidone solution has not become widely applied, as it might cause local inflammation [123] and reduced expression of endometrial progesterone receptors, hindering fertility [124].

Accumulation of exudate within the uterine lumen can inactivate or dilute the infused antibiotic [117], thus removal of the exudate would increase antibiotic efficacy. Hence, the possibility of an animal to effectively expel and clear intrauterine fluid has been used to indicate mares, which may show a higher susceptibility to endometritis subsequent to mating [125,126,127]. With regard to the clinical use of specific antibiotics, amikacin is considered to be the antibiotic with the broadest spectrum of activity against uterine pathogens, as well as presenting excellent activity against *Ps. aeruginosa* [107]. Gentamicin is often used for intrauterine administration; the antibiotic needs to be buffered with bicarbonate before infusion [107]. In cases of administration of penicillin and ampicillin, it should be taken into account that these antibiotics are inactivated in pH ranges below 5.5 or over 8.0 [107]. The use of tetracyclines in horses should be carefully assessed against the possible risk of various important adverse reactions, among them severe colitis [128,129]. Given this, as well as the isolation of tetracycline-resistant pathogens from samples of equine origin, clinicians should carefully consider the use of this antibiotic [130,131]. The use of combinations of antibiotics (i.e., neomycin plus polymyxin B plus crystalline benzylpenicillin) affords a broad-spectrum activity against many common pathogens, as well as specific anaerobe bacteria (e.g., *Bacteroides fragilis*) and has also been reported for clinical application [107]. However, it is noted that in the European Union that there are no preparations licenced for intrauterine administration to equids, hence clinicians should apply the cascade rule when considering such a therapeutic approach.

### 4.3. Antibiotic Use in Male Genital Infections

The treatment regime of penile and preputial infections caused by opportunistic pathogens usually consists of using disinfectants, as systemic antibiotic administration is often unrewarding [132]. When artificial insemination is used, penicillin G, streptomycin, amikacin or gentamicin are routinely used in semen extenders [133]. In clinical cases of orchitis or epididymitis, treatment includes the use of systemic antibiotics, based on semen culture and in vitro susceptibility testing of isolated bacteria [134]. In the rare cases of seminal vesiculitis, treatment with systemic antibiotics is considered to be difficult due to the impaired diffusion across mucosal cell borders into the seminal plasma [107]; there is a report in the international literature describing transurethral administration of antibiotics to the seminal vesicle [70]. If local administration is not feasible, then systemic administration needs to be considered; potentially suitable antibiotics for this include macrolides (e.g., erythromycin) and enrofloxacin.

### 4.4. Antibiotic Use in Mammary Infections

Treatment of equine mastitis should be based on systemic administration of antibiotics [106,135]. Broad-spectrum antimicrobials, e.g., trimethoprim–sulphonamides, can be administered initially until the results of bacterial identification and susceptibility testing become available [106], which would direct appropriate antibiotics for administration. The use of intramammary tubes, which are licenced against bovine mastitis can be considered [103], but their administration may be controversial, as these preparations are not licenced for equids and lack approved withdrawal periods for these species. Anatomical differences between bovine and equine teat and mammary gland may result in incorrect administration of such products and, ultimately, in reduced efficacy [88].

## 5. Antibiotic Resistance as a Limiting Factor in Treatment

**Figure 1:**
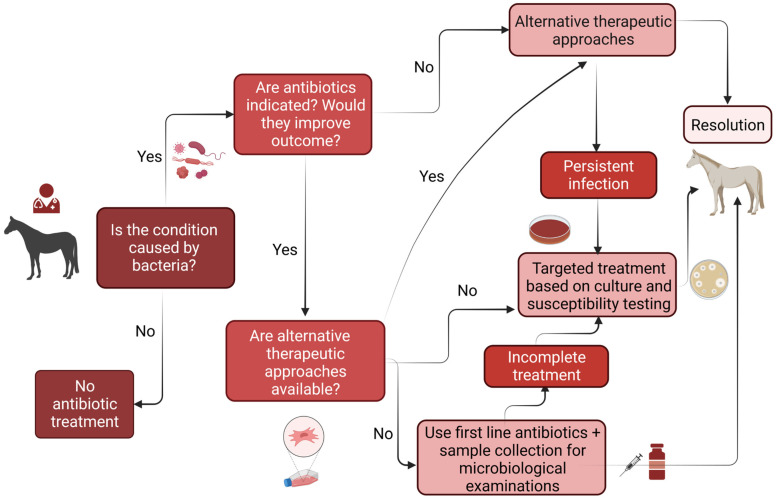
Diagram of suggested actions in the face of possible antibiotic resistance in reproductive infections of equids (created with BioRender.com, accessed 14 March 2023).

Antibiotic resistance of bacteria isolated from equine reproductive organs has been reported for animals with fertility problems, as shown in studies performed in various countries in Europe (France [10,130], Germany [136], Italy [42,137], Slovakia [43], Sweden [20], Turkey [138], United Kingdom [139]) and elsewhere (Brazil [140], Canada [141], India [142], United States of America [143]). Antimicrobials are commonly used to treat empirically reproductive disorders. One may suggest that continuing problems are the consequences of inappropriate treatment regimes applied in the animals. The expansion of antibiotic resistance among equids, additionally to the clinical implications for the animals, is also important within the broad concept of One Health as resistant isolates can be transmitted to other animal species and people [144,145]. A diagram of suggested actions in the face of possible antibiotic resistance is shown in Figure 1.

Emergence of methicillin-resistant staphylococci, especially in equine hospitals, is leading to increased monitoring for these bacteria because of the difficulties in their treatment and the importance for public health [6,146,147,148,149]. Methicillin resistance often occurs as the result of carriage of a SCCmec gene cassette [6,150].

Haemolytic and non-haemolytic *E. coli* isolates recovered from horses were found to be resistant to *β*-lactams, aminoglycosides, tetracyclines, fluoroquinolones and potentiated sulphonamides [43,151,152,153,154,155,156,157,158]. A common resistance-inducing mechanism in *E. coli* is the ability to produce extended-spectrum *β*-lactamases [9,159,160]. *β*-lactamase producing strains have also been detected in other Enterobacteriacae, e.g., *Klebsiella* spp., suggesting a spread among different bacterial species, which may complicate antibiotic stewardship decisions [161].

*Acinetobacter* spp. isolates recovered from jennies were found to possess specific resistance mechanisms, including class 1 integrons that contained multiple aminoglycoside resistance and specific carbapenemase genes [6,162]. It is noted that as carbapenems are considered critically important antibiotics for human medicine [163], their use in equids should be limited strictly to critical cases. Finally, endometritis associated with an antibiotic-resistant isolate of *Ps. aeruginosa* has been reported in Australia [164].

Another significant issue, of importance in equine clinical work, is the potential role of semen extenders in antibiotic resistance. A recent report indicated that isolates of *Acinetobacter* spp. and *Klebsiella* spp. recovered from mares in which artificial insemination was used showed antibiotic resistance more frequently than similar bacteria obtained from non-inseminated animals [68].

## 6. Approaches to Circumvent Antibiotic Resistance

**Figure 2:**
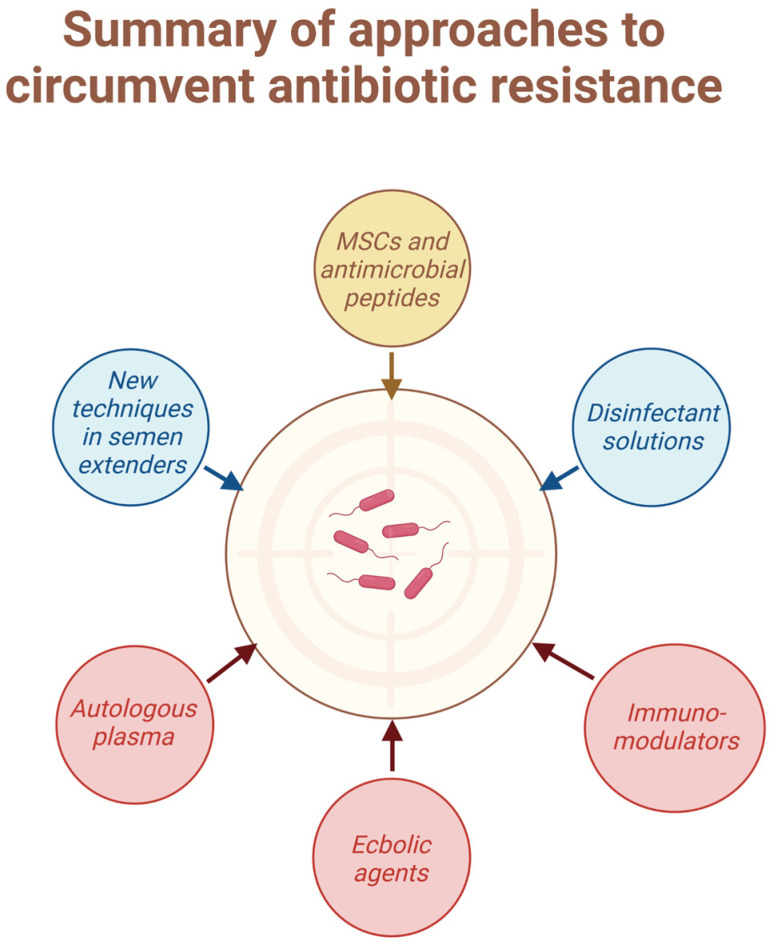
Approaches other than administration of antibiotics that can be employed to circumvent antibiotic resistance in reproductive medicine of equids (created with BioRender.com; accessed 14 March 2023).

Failure of equine clinicians to address antibiotic resistance will inevitably generate persistent infections and might result in suboptimal reproductive performance, leading clinicians to face increasing challenges. Any attempt to obviate the excessive and inappropriate use of antibiotics should be encouraged. Moreover, the use of therapeutic protocols that employ other approaches should also be considered (Figure 2).

### 6.1. Administration of Autologous Plasma

The rationale for treatment with autologous plasma (i.e., plasma with high concentration of thrombocytes with large amount of proteins, resulting in enhancement of an animal’s natural defence response) has been based on the administration of immunoglobulins and complement to support treatment of bacterial endometritis in mares [165]. In an in vitro study, the potential antimicrobial properties of platelet lysate were highlighted against various Gram-positive or -negative bacteria and were found to be concentration-dependent [166]. Under clinical conditions, improvement of fertility of mares with persistent post-breeding endometritis has been reported after administration of autologous plasma [167].

### 6.2. Administration of Ecbolic Agents

The traditional treatment of many common reproductive disorders in female equids includes the application of uterine lavage and the use of ecbolic agents (or uterotonics), which are pharmacological substances employed for the initiation of contractions or increased tonicity of the uterus, for the induction of parturition and/or for the reduction of post-partum haemorrhage [168], which aim to adjunct uterine clearance, especially in cases of impaired myometrial contractility. Studies have suggested that the above combination can show the same efficacy as antibiotic administrations against post-mating uterine infections in mares [113,126]. Other studies, in which concurrent uterine lavage and administration of prostaglandin F_2_*α* was compared to intrauterine administration of penicillin against *S. equi* subsp. *zooepidemicus* uterine infection, did not show differences in efficacy between the two regimes [107]. Hence, the author postulated that if treatment with prostaglandin F_2_*α* would be initiated early, antibiotic administration may not be necessary [107].

Other ecbolic agents commonly employed include oxytocin or cloprostenol. Oxytocin increases the risk for development of colic-type clinical signs in horses after administration [169,170]. Therefore, as it is often important of using it in emergency reproductive disorders (e.g., retention of foetal membranes), it should be administered at low doses, which minimise that risk [171]. A potential benefit of oxytocin administration after mating or artificial insemination refers to the increase of contractions of uterine muscles to eliminate excessive spermatozoa, as well as bacteria that might have invaded into the uterus at that time. Oxytocin is used because of its contractile action on the myometrium that would accelerate uterine involution and mucosal degeneration [121]. However, at later stages during gestation, the increased uterine contractility may cause adverse effects to the foetus borne, hence caution must be applied when administering to mares the correct oxytocin dose according to specific situations [172].

Moreover, it is noteworthy that, in another study, Li et al. [173] reported that administration of oxytocin and uterine lavage alone may not be as effective as presumed. In their work, these authors indicated that concurrent administration of immunomodulatory agents and blood cell treatments would have better results in the treatment of endometritis caused by streptococci [173].

In any case, the short half-life of oxytocin requires frequent repeated administration, whilst cloprostenol induces uterine contractions of longer duration [114]. As an alternative, the administration of carbetocin, a long-acting oxytocin analogue with a long half-life after intravenous injection, has also been reported [174,175].

### 6.3. Administration of Immunomodulators

The immune response of the endometrium of mares has been extensively investigated [176,177]. A positive effect of corticosteroid administration on the uterine immune response has been reported in several studies [176,177,178,179], which may ultimately improve fertility of animals. For example, administration of dexamethasone at breeding was found to improve pregnancy rates in mares with a history of post-mating endometritis [180]. Moreover, higher pregnancy rates were recorded in mares with a history of the disorder, after administration of prednisolone at breeding [177].

### 6.4. Use of Alternative Techniques in Semen Extenders

The addition of antibiotics in semen extenders (as a means to limit bacterial growth and multiplication therein) may trigger changes in susceptibility/resistance patterns of vaginal bacteria, subsequent to the exposure to semen extenders [181]. Hence, alternative therapeutic approaches to conventional antibiotics should be employed for the same objective. For example, the microfiltration of seminal plasma through a syringe prefilter has been found to reduce its bacterial load [182]. In addition, colloid centrifugation has been reported as a simple method to separate spermatozoa from bacteria in an ejaculate [183]. Colloid centrifugation is a semen preparation technique, whereby a sub-population of motile spermatozoa with normal morphology, intact membranes and good chromatin integrity are separated from the ejaculate, including the seminal plasma, by centrifugation through a colloid [183].

### 6.5. Use of Disinfectant Solutions

Treatment for infections of the genital system of male equids, caused by *Ps. aeruginosa* or *Kl. pneumoniae*, can be performed by means of daily application of disinfectant solutions, e.g., iodine-based surgical scrubs, hydrochloric acid diluted solutions or sodium hypochlorite bleach solutions, on the external genitalia of the animals [107].

### 6.6. Use of Equine Mesenchymal Stem Cells and Antimicrobial Peptides

Studies have discussed the immunomodulatory nature of mesenchymal stem cells and the role of antimicrobial peptides, providing a potential alternative to use of antibiotics, by expressing antibacterial proteins, e.g., lipocalin-2 [184]. Harman et al. [185] have assessed the potential antibacterial activity of these cells and reported that secreted antimicrobial peptides successfully inhibited growth of bacteria isolated from skin wounds of horses.

## 7. Concluding Remarks: Perspectives and Future Directions

Antibiotic resistance is an important global public health threat [186]. Improving antimicrobial stewardship can be achieved through an array of measures, as well as by means of various approaches to maintain the efficacy of antimicrobials in human, animal and agricultural settings. In this respect, initiatives are being taken by many organisations and authorities, in order to control antibiotic resistance.

Among these, regulatory and voluntary initiatives aim to decrease the dissemination of antibiotic-resistant bacteria to animals, as well as to people, given that recent studies have linked horses as a potential reservoir of a variety of pathogens, which may be transmitted to people [187]. For example, methicillin-resistant *S. aureus* from infections of horses have been found to disseminate often to veterinarians in Germany [147]; moreover, Albert et al. [145] have recently described the first direct transmission of a *mecC*-carrying methicillin-resistant *S. aureus* between a horse and the attending veterinarian. Examples of regulatory interventions taken to control antimicrobial resistance may include strict regulations for licencing, prescribing and marketing of antibiotics [188].

Awareness regarding antibiotic resistance in equine reproductive medicine would increase, as we would recognise the multifaceted problem of resistance. This practice may lead to undesirable microbial imbalance and can favour the acquisition of antibiotic resistance. It is imperative for equine clinicians to understand local patterns of antibiotic resistance when considering and developing treatment regimes [189]. Continued engagement of equine clinicians with the current trends on novel alternative approaches to treat genital infections is essential in order to address this rising threat for the health and welfare of animals, as well as within the One Health perspective.

With regard to work in equids, specific initiatives have been set up. Examples include the BEVA Toolkit in the United Kingdom [190] and the development of a group for equine veterinary work within the European network for the optimum use of antibiotics [191], which aim to guide veterinarians active in equine practice in the correct use of antibiotics. These initiatives show directions for future actions and can be used as examples for furthering such actions internationally within the context of One Health.

This approach, additionally to control of dissemination between animals and people, also involves the monitoring of antibiotic-resistant bacteria or resistance genes to various environmental matrices; these may refer to waste, surface waters, agricultural runoff and water for use in aquaculture, and should not be ignored, further shaping the directions for controlling the problem as much as possible. In recent relevant studies, the environment–animal sharing of resistance has been found to have hindered the impact of interventions for the reduction of antibiotic consumption in animals and to have affected the epidemiology of infections caused by resistant bacteria [192].

## Figures and Tables

**Table 1:** Examples of commonly applied antibiotic administration regimes in cases of genital infections of equids.

Antibiotic (Dose Rate)	Route of Administration	Comments
Amikacin sulphate (10 mg kg^−1^ per 24 h)	Systemic (intravenous (i.v.) or intramuscular (i.m.)	Good efficacy against uterine infections caused by Gram −ve bacteria. Resistance by staphylococci and streptococci. Inactivation in purulent material. Recommendation for administration in infections caused by gentamicin-resistant bacteria. Nephrotoxicity.
Amikacin sulphate (2 g)	Intrauterine	Buffering with bicarbonate or dilution with large volume of normal saline.
Ampicillin sodium (20 mg kg^−1^, per 6–8 h)	Systemic (i.v. or i.m.)	Improved efficacy over penicillin against Gram −ve bacteria.
Ampicillin (1 g)	Intrauterine	Effective against Gram +ve bacteria.
Benzylpenicillin (22,000 IU kg^−1^ per 4–6 h)	Systemic (i.v.)	Antibiotic of choice against streptococcal infections. Excellent efficacy against anaerobes (except *Bacteroides* spp.). Possibility for combination with aminoglycosides for broad spectrum coverage.
Benzylpenicillin	Intrauterine	Inactivation in solutions with pH < 5.5 or pH > 8.0. No mixing with gentamicin, sulphonamides or sodium bicarbonate. Use should be guided by bacteriological culture, cytology or ultrasonographic findings.
Ceftiofur sodium or ceftiofur crystalline free acid (1.1–2.2 mg kg^−1^ per 12 h)	Systematic (i.v. or i.m.)	Avoidance of use, in alignment with recommendations for keeping 3rd generation cephalosporins (e.g., ceftiofur) as reserve drugs [95]. Possible swelling at injection site.
Enrofloxacin (5–7.5 mg kg^−1^)	Systemic (i.v.) or *per os*	Broad spectrum activity. Avoidance of use, in alignment with recommendations for keeping 3rd generation fluoroquinolones as reserve drugs. Use only based on results of bacterial culture and susceptibility testing. Generally, avoidance of use in horses < 4 years of age and during pregnancy. Synergism with *β*-lactams and aminoglycosides. Intrauterine administration of the licenced product for administration to cattle: not recommended (association with development of necrosis) [96].
Gentamicin (adult animals: 6.6 mg kg^−1^ per 24 h)	Systemic (i.v.)	Potential development of nephrotoxicity or ototoxicity. Development of muscular discomfort after intramuscular administration [97].
Gentamicin (1–2 g)	Intrauterine	Irritation to endometrium or induction of depigmentation of vulvar skin if administered without buffering (performed with an equal volume of 7.5% sodium bicarbonate and dilution into 200 mL normal saline). Recommendation for administration in infections by Gram −ve bacteria. Inactivation by purulent material. Synergistic action with drugs that interfere with cell wall synthesis, e.g., penicillin [98].
Oxytetracycline (6.6 mg kg^−1^ per 12 h)	Systemic (slow i.v.)	Broad-spectrum antibacterial activity. Good spectrum of activity against anaerobic bacteria, but variable against *Bacteroides* spp. and *Clostridium* spp. Adverse effects: hypotension and collapse after rapid intravenous administration, renal tubular necrosis after administration of high doses, colitis and irritation after extravascular administration [99].
Procaine penicillin (20,000–25,000 IU kg^−1^)	Systemic (i.m.)	Efficacy against infections by *β*-haemolytic streptococci, anaerobic organisms (bar *Bacteroides fragilis*) and some Gram −ve bacteria (e.g., *Actinobacillus* spp. and *Pasteurella* spp.). Time dependent action. Long-acting formulations inappropriate for horses. Adverse effects: possibly hypersensitivity reactions (urticaria, anaphylaxis, immune mediated haemolytic anaemia) [100].
Procaine penicillin (1.2 g)	Intrauterine	
Trimethoprim/sulfamethoxazole (30 mg kg^−1^ per 12 h)	Systemic (slow i.v.) or *per os*	Inactivation in purulent material [101]. Concurrent administration of penicillin can act antagonistically to sulphonamides. Drugs are irritant if administered intramuscularly or by the intrauterine route [102].

**Table 2:** Examples of commonly applied antibiotic administration regimes in cases of mammary infections of equids.

Antibiotic (Dose Rate)	Route of Administration	Comments
Amoxicillin	Intramammary	Administration of products for intramammary administration licenced for cattle under provisions for ‘off-label’ use. Choice should depend on the results of culture and sensitivity examinations. Administration of ‘dry cow’ products may provide long-standing antibiotic activity after the end of therapeutic administration [88,103].
Ceftiofur crystalline	Systemic	Empirical treatment regime with questionable efficacy [96].
Ceftiofur hydrochloride	Intramammary	Administration of products for intramammary administration licenced for cattle under provisions for ‘off-label’ use. Choice should depend on the results of culture and sensitivity examinations.
Cefquinome	Intramammary	Administration of products for intramammary administration licenced for cattle under provisions for ‘off-label’ use. Choice should depend on the results of culture and sensitivity examinations.
Gentamycin	Intramammary	Intramammary administration of a product available in pharmaceutical form for injectable administration [104].
Trimethoprim/sulfamethoxazole (30 mg kg^−1^)	*Per os*	Empirical treatment regime, often used until results of bacteriological examinations (bacterial identification and sensitivity testing) become available [105,106].

## Data Availability

Not applicable.
